# Exploring Factors Link to Teachers’ Competencies in Entrepreneurship Education

**DOI:** 10.3389/fpsyg.2020.563381

**Published:** 2020-11-05

**Authors:** Yangjie Huang, Lanyijie An, Lanying Liu, Zelin Zhuo, Peng Wang

**Affiliations:** ^1^Institute of China Innovation and Entrepreneurship Education, Wenzhou Medical University, Wenzhou, China; ^2^Institute for Advanced Study of Educational Development in Guangdong-Hong Kong-Macao Greater Bay Area, South China Normal University, Guangzhou, China

**Keywords:** entrepreneurship education, educational evaluation, competency, teacher training, education quality

## Abstract

To improve the quality of entrepreneurship education, this study explored factors linked to teachers’ competencies in entrepreneurship education in China. A regression analysis based on 12,596 teachers’ questionnaires showed that the competencies of teachers in entrepreneurship education had three dimensions. The factors linked to teacher’s competencies were professional training, new modes of teaching, entrepreneurial practice, entrepreneurial culture, and policy guarantee. At the same time, the influence mechanism between teachers’ overall competency and factors is explored. For a higher quality of entrepreneurship education, it is necessary to adopt new modes of teaching, pay attention to teachers’ careers, and improve the evaluation and recruitment mechanism for teachers. Our findings provide novel insights by exploring factors linked to teachers’ competencies, extending understanding on improving the quality of entrepreneurship education, and enriching the entrepreneurship education literature by adding new empirical evidence from China.

## Introduction

Entrepreneurship education (EE) is growing worldwide (Kuratko, 2005; Siegel et al., 2007; Katz, 2008). EE in China has developed rapidly since 2015. In September 2018, the State Council of the People’s Republic of China issued opinions on promoting high-quality development of innovation and entrepreneurship and creating an upgraded version of mass entrepreneurship and innovation. Teachers of EE play a key role in improving the quality of personnel training and accelerating the development and upgrading of innovation and entrepreneurship (Seikkula-Leino et al., 2010; Teerijoki and Murdock, 2014; Ruskovaara and Pihkala, 2015; Ruskovaara et al., 2015). Teacher competencies largely determine EE outcomes (Martin et al., 2013).

Innovation and EE is the trend of “the times” (de la Fuente et al., 2018; Sánchez-García et al., 2018). EE aims to cultivate students’ entrepreneurial ability and thinking and to promote students’ successful entrepreneurship (Wu and Wu, 2017). Fayolle et al. (2019) showed that the effects differ depending on trainees’ personal characteristics and training strategies, which can have varied impacts on learning processes and results. Entrepreneurship learning is conducive to improving students’ willingness to start a business and enterprise growth performance (Liu et al., 2019; Wu W. et al., 2020). The development of EE cannot be separated from the development of entrepreneurial teachers. EE requires teachers to teach students knowledge, skills, and mindset through various teaching methods (Yuan and Wu, 2020). Due to the particularity of EE, entrepreneurship teachers, compared to ordinary teachers, also need to emphasize entrepreneurship skills. That is, the competencies structure of entrepreneurship teachers includes attitude, knowledge, and entrepreneurship skills (Huang et al., 2017). However, the dearth of highly capable EE teachers is the main obstacle at colleges and universities (Dahl, 2019). This study provides novel insight into EE, serves as an initial benchmark in the field, and provides some useful implications for EE practicing managers and teachers.

## Literature Review

### Research on Competency Theory

**FIGURE 1:**
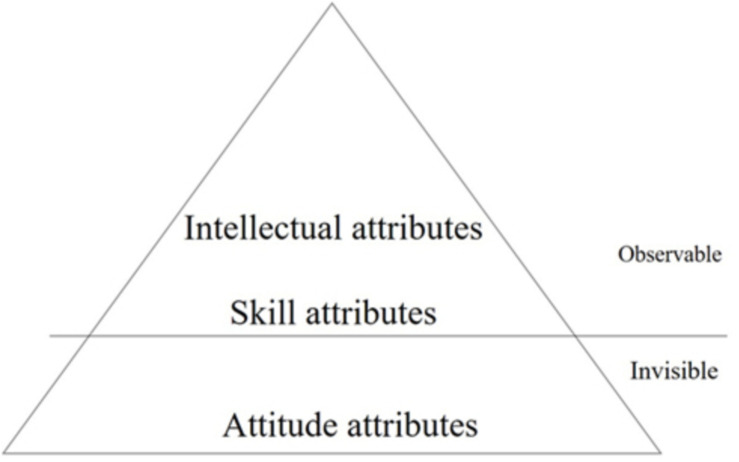
Competency model.

Competency is a comprehensive concept that combines both generic and specific aspects (see Figure 1) and is extrapolated from performance and observable activity to implied attributes (Gonczi, 2013). Some studies believe that training teachers’ professional competency and teachers’ positive attitude toward classroom learning can improve teachers’ complete participation rate in classroom learning (Jhang, 2019). In Kuivila’s research, teacher competency included leadership and management competency, evidence-based practice competency, subject competency, ethical competency, pedagogical competency, collaboration competency, internationalization competency, and continuous professional development competency (Kuivila et al., 2020). Some scholars believe that the concept of mathematics teachers’ competency includes the subject characteristics and common aspects of mathematics teachers’ knowledge, skill, and belief (Blömeke et al., 2020). In this study, for entrepreneurial teachers, subject specificity refers to their entrepreneurial competency while the common aspects refer to their teaching competency. There are two definitions of competency in the existing literature: trait theory (Fleeson and Jayawickreme, 2015) and behavior theory (Ajzen, 1991).

For instance, self-confidence, risk-taking behavior, internal locus of control, need for achievement (Vodã and Florea, 2019), and innovativeness, etc., are all aspects of trait theory. Hayat and Amer (2013) found that student perception can be explained using the innovativeness, proactiveness, and risk-taking attributes of a teacher in Finland and Pakistan. Hameed et al. (2016) found a strong need to revise the curriculum of higher education institutions in the UAE to develop self-confidence, locus of control, and risk-taking propensity among students. Winkler (2014) found that EE is a dynamic field of inquiry that considers the triadic reciprocal relationship of the personal, behavioral, and environmental factors of entrepreneurial learning. Hence, it also contains a lot of behavior-related competency elements, such as competency to identify and grasp opportunities, integrate resources, propose ideas, implement innovation, set up entrepreneurial teams, operate, and manage. Further, Wu et al. (2018) suggested that information and communication technology can be used to enhance the effectiveness of traditional teaching methods and competency training in EE. An increasing number of scholars are using competency theory to study universities and teachers, for example, using the teacher competency training system to improve competency for education for sustainable development (Weng et al., 2020) and developing a competency-based framework for teachers’ entrepreneurial behavior (Van Dam et al., 2010), teachers’ entrepreneurial competencies (Peltonen, 2015; Stettiner et al., 2015), the capacity building of teachers of innovation and EE in universities (Trauth et al., 2015), and research on teacher competency and instructional quality (Blömeke et al., 2020).

### Research on the Structure of Teacher Competencies in EE

The European Union (EU) described the basic connotation of “entrepreneurial teacher” from the perspective of trait theory and summarized several characteristics (Redford, 2015), including the need to love their own career and need to have an optimistic and positive spirit. Shane believed that the two main competencies of entrepreneurs are exploring and developing opportunities (Eckhardt and Shane, 2003; Shane, 2009; Shane and Dolmans, 2015). Alvarez (Alvarez and Busenitz, 2007) argued that entrepreneurial capacity was the competency to reintegrate resources. Schelfhout et al. (2016) determined a behavior-oriented and education-oriented entrepreneurial competency measurement tool and proposed that the sub-capabilities of entrepreneurial competency can be further subdivided into 21 various behavioral indicators. The EU has summarized three aspects of entrepreneurial teachers based on their action characteristics: first, entrepreneurial teachers are good at listening and can find good ideas from conversations; second, they are proactive and good at selling their own ideas to others; and third, they cultivate students’ enthusiasm for creation, growth, and learning.

Therefore, teachers’ competencies in EE refer to the comprehensive qualities of attitude, knowledge, and skills needed in colleges and universities to successfully complete their related work.

### Research on the Factors Linked to Teachers’ Competencies in EE

There is considerable literature on the impact of entrepreneurship on students. Fang and Chen (2019) explored the entrepreneurial intentions of science and engineering students in China. Pauceanu et al. (2019) explored what determinants influence students to start their own business in United Arab Emirates Universities. Yi (2017) explored the relationship between internship quality, entrepreneurial desirability, entrepreneurial feasibility, and entrepreneurial intention among graduating engineering students of research universities in China. Yin suggests building sustainable core competency through knowledge management (Yin et al., 2019).

The influencing factors for teachers’ competencies in EE mainly include two dimensions: internal and external factors. In recent years, scholars have called for the construction of specialized innovation and EE faculty through institutional innovation and other measures in Chinese universities (Fu, 2015; Shen, 2015; Huang et al., 2017). They put forward some strategies such as establishing EE discipline, offering professional degree education, setting up full-time teaching posts, establishing an incentive mechanism, breaking system flow barriers, and improving the entrepreneurial ecosystem.

Therefore, based on the empirical research of 12,596 teachers nationwide, our research questions were:

(1) What is the structure of teachers’ competencies in EE?
(2) What are the factors linked to teachers’ competencies?
(3) How can teachers’ competencies be improved?

## Methodology

### Participants and Procedure

#### Model

Based on the above literature and grounding theory (Bowen, 2005), this paper interviewed 30 Chinese EE teachers, such as Xiaozhou Xu (Ni and Xu, 2016), Zhanren Wang, and Weihui Mei, and identified five factors and 17 secondary indicators. The internal factors included professional training, new modes of teaching, and entrepreneurial practice. The external factors included entrepreneurial culture and policy guarantees.

Professional training included three secondary indicators, mainly related to the professional training of EE teachers. New modes of teaching included three secondary indicators, which mostly involve the innovation of entrepreneurial teachers’ teaching methods (Hsu, 2019; Kuo and Hsu, 2020; Wu L. et al., 2020). Entrepreneurial practice included three secondary indicators, mainly involving teachers’ own entrepreneurial practice experience. Entrepreneurial culture included three secondary indicators, which mainly relate to the role of teachers in EE. Policy guarantee included five secondary indicators, mainly related to the policy in the protection and incentive of EE teachers.

#### Data Collection

The questionnaire investigated teachers using a total of 29 items obtained from an extensive literature review on EE. We conducted a rolling survey from September 15, 2018 to January 18, 2019. Finally, 13,120 questionnaires were collected, among which 12,596 were valid questionnaires, accounting for 96.01% of the total. The questionnaires were distributed to 596 colleges and universities in 30 provinces of those, 524 questionnaires were invalid, due to a too short answering time and invalid school name, and these were eliminated (see Table 1). Specifically, there were 12 secondary indicators: entrepreneurial identity, entrepreneurial will, entrepreneurial spirit, pedagogy related knowledge, teachers’ professional knowledge of their own discipline, venture capital knowledge, entrepreneurial knowledge, teaching organization skills, entrepreneurial practice skills, opportunity identification skills, opportunity development skills, and management and operation skills (see Table 2).

**TABLE 1:** Basic information of sampling teachers (*N* = 12596).

Items		Frequency	Percentage
Gender	Male	5498	43.6
	Female	7098	56.4
Teachers’ age	30 years old and under	4927	39.1
	31–35 years old	2953	23.4
	36–40 years old	2643	21.0
	41 years old and above	2073	16.5
The highest degree	Bachelor’s degree	2447	19.4
	A master’s degree	6800	54.0
	Dr.	1843	14.6
	other	1506	12.0
Type of school	Double first-class universities*	1241	9.9
	General university	6067	48.2
	Independent college	891	7.1
	Higher vocational colleges	3278	26.0
	Others	1119	8.8

*Double first-class universities: “Double-First Class” means world-class universities and first-class disciplines. The plan, also known as the “Double-First Class” initiative, began in 2017.

**TABLE 2:** structure of teachers’ competencies of entrepreneurship education.

Competency structure	Secondary indexes	Mean	SD
Attitude attributes	C1 teachers agree with the idea of entrepreneurship education	3.92	0.877
	C2 teachers have entrepreneurial will	3.96	0.894
	C3 teachers have entrepreneurial spirit	4.01	0.879
Intellectual attributes	C4 teachers have knowledge of pedagogy	3.97	0.853
	C5 teachers have entrepreneurial knowledge	4.07	0.842
	C6 teachers have professional knowledge of the subject	4.05	0.871
	C7 teachers have knowledge of venture capital	3.97	0.846
Skill attributes	C8 teachers have teaching organization skills	4.04	0.842
	C9 teachers have entrepreneurial practical guidance skills	4.12	0.838
	C10 teachers have entrepreneurial opportunity recognition skills	4.11	0.853
	C11 teachers have entrepreneurial opportunity development skills	4.06	0.849
	C12 teachers have project management, operation, and coordination skills	4.10	0.894

The questionnaire used a five-point Likert scale, 5 meaning “I couldn’t agree more” and 1 meaning “Highly disagree” to evaluate the competencies of teachers, we asked, “Based on your actual experience, evaluate the competencies scale of entrepreneurship education teachers” (see Table 2). To evaluate the factors linked to teachers’ competencies in EE, we asked, “Based on your actual experience, evaluate the scale of factors for teachers’ competencies improvement in entrepreneurship education” (see Table 3).

**TABLE 3:** Factors link to teachers’ competencies of entrepreneurship education.

Influence factors		Mean	SD
Professional training (PT)	M2 encourages teachers to participate in entrepreneurship training	4.15	0.830
	M5 attaches importance to entrepreneurship education in pre-service teacher education	3.98	0.849
	M17 provides scientific career planning for the professional development of entrepreneurial teachers	4.08	0.817
New modes of teaching (NMT)	M4 encourages teachers to integrate professional courses with entrepreneurship education	4.16	0.828
	M7 focuses on active learning and experiential learning	4.13	0.821
	M18 attaches great importance to the theoretical and practical research of teachers’ innovation and entrepreneurship education	4.11	0.809
Entrepreneurial practice (EP)	M6 encourages teachers to take part-time jobs in SMEs	4.04	0.865
	M13 teachers’ original entrepreneurial experience	3.99	0.828
	M14 sets up network communication groups of entrepreneurial teachers in the whole province or the whole country	3.99	0.838
Entrepreneurial culture (EC)	M10 defines the role of teachers in entrepreneurship education	4.15	0.806
	M11 explores and sets up successful entrepreneurship models for teachers	4.06	0.846
	M12 creates a strong culture of innovation and entrepreneurship	4.16	0.825
Policy support (PS)	M8 improved the evaluation and recruitment standards and performance appraisal standards for teachers of entrepreneurship education	4.10	0.861
	M9 improves the income distribution mechanism of scientific and technological achievements	4.12	0.825
	M15 provides policy guarantee for teachers who have left their posts to return to their posts	4.08	0.835
	M16 provides policy support for the promotion of professional titles of teachers who have left their posts for entrepreneurship	4.08	0.843
	M19 design policy provides time guarantee for teachers to guide students to start their own businesses	4.15	0.809

#### Population-Sample

The survey objects and requirements of the teacher’s questionnaire were leading cadres, administrative personnel, and professional teachers related to EE.

Specifically, it included the following categories:

(1) Teachers of various types of innovation and entrepreneurship courses.
(2) Mentors for students’ various entrepreneurship competitions, for students’ entrepreneurship projects such as pioneering parks and science and technology parks, and for students’ entrepreneurship scientific research projects, innovation experiments, and publishing papers.
(3) Entrepreneurship management personnel at all levels of the university, including university leaders, department leaders, and other entrepreneurship management personnel.
(4) Teaching management personnel, ideological and political workers (students’ engineering, youth league committee, counselors, etc.) of the school and secondary colleges, and personnel department and logistics management personnel (in Chinese universities, since ideological and political workers mostly hold concurrent educational positions, such as counselors, they were also included in our survey).
(5) Teachers engaged in EE and scientific research.

### Scale Reliability and Validity Test

The teacher questionnaire used in this paper is based on domestic and foreign journal literature, comparing and analyzing existing questionnaires on EE, and integrating in-depth semi-structured interviews and analyses of many experienced EE teachers. After the first draft of the questionnaire was generated and distributed to several scholars in the field of EE to modify, the team tested the questionnaire on a total of 98 colleges and universities across the country, including a preliminary verification questionnaire item validity and reliability and whether it was enough to explain this topic in the theoretical model. Finally, the finished questionnaire was created and distributed online to 596 colleges and universities across China.

Reliability refers to the degree of consistency or reliability of the test results. Corrected Item Total Correlation (CITC) and Cronbach’s alpha were calculated to test the reliability of the scale in this research. A reliability test was conducted by calculating the CITC of each measurement item. If the value was less than 0.5 (Zijlmans et al., 2019), the index was deleted. Meanwhile, Cronbach’s alpha coefficient was calculated. Cronbach’s alpha is the most commonly used method for statistical reliability measurement. It is usually evaluated between 0 and 1. If the alpha coefficient is ≥0.7 (Zijlmans et al., 2019), the reliability of the indexes is acceptable. The CITC minimum value of entrepreneurial education teachers’ competency scale was 0.692, Cronbach’s alpha was 0.952, and the Cronbach’s alpha coefficient value after deleting all the observed variables in the table was smaller than the original scale’s alpha value, indicating that the scale had high reliability. Similarly, the reliability test was carried out on the factors linked to teachers’ competencies in EE. In the Professional Training dimension, the Cronbach’s alpha coefficient was 0.827, the CITC minimum value was 0.682, and Cronbach’s alpha coefficient value after deleting all the observed variables in the table was smaller than the original scale’s alpha value indicating that the scale also had high reliability. Similarly, the Cronbach’s alpha coefficient and the CITC minimum value were 0.853 and 0.707, respectively, in the new modes of teaching dimension; 0.805 and 0.627, respectively, in the entrepreneurial practice dimension; 0.882 and 0.767, respectively, in the entrepreneurial culture dimension; 0.906 and 0.724, respectively, in the policy support dimension. All the reliabilities of the indexes were acceptable.

Validity testing is used to verify whether the scale design is reasonable, which is done by exploratory factor analysis. KMO (Kaiser–Meyer–Olkin) and Bartlett tests were carried out on the scales of teachers’ competencies structure and influencing factors, and the results showed that KMO was greater than 0.7. The significance probabilities of Bartlett’s sphere test were all 0.000, indicating that the data were correlated, which was suitable for factor analysis.

### Entrepreneurship Policy

Guided by the scope of this study, entrepreneurship policy was measured according to four criteria: (1) The state has reduced or exempted taxes on businesses founded by self-employed college students. (2) Local governments have simplified the application process for university students to register their businesses. (3) The school provides start-up funds for business (interest-free loans). (4) Society offers free training to guide entrepreneurship. Responses to these prompts were scored a maximum of five points. The Cronbach’s alpha coefficient was 0.960.

## Research Results

### Structure and Factors Linked to Teachers’ Competencies

All the questions on teachers’ competencies in EE in Chinese universities was evaluated on a 5-point scale, with 5 being “I couldn’t agree more” and 1 being “highly disagree” (the results are shown in Table 2 below). The mean value of all indicators was around 4.0, indicating that the sampled teachers thought that these indicators were relatively important, while the five indicators of skill attributes (C8–C12) were all higher than 4.0, indicating that the sampled teachers thought skill attributes were a relatively important part of the structure of teachers’ competencies. Among these, “C9 – Teachers have entrepreneurial practice guidance skills” was the most important.

Similarly, combined with the actual experience of teachers, this paper evaluated the factor scale of improvement in teachers’ competencies. The average value of each index was around 4.0, indicating that the sampled teachers thought these indicators were relatively important. Among them, the three indexes of “new modes of teaching” (M4, M7, and M18) were all higher than 4.1, indicating that the new teaching factors had a higher impact on the improvement of teachers’ competency than the other three factors. The sampled teachers thought that encouraging teachers to deeply integrate professional courses with EE and create a strong entrepreneurship culture were the most important indicators among the influencing factors, with an average score of 4.16. Attaching importance to pre-service teacher education had the lowest score, with an average of 3.98.

### Multiple Regression Results

The correlation analysis results of the factors affecting the overall competency of teachers are shown in Table 4 below, which shows that further regression can be achieved. The results of multiple regression analysis are summarized in Table 5. SPSS 25.0 was used for multiple linear regression. The independent variables were professional training, new modes of teaching, entrepreneurial practice, entrepreneurial culture, and policy support. The dependent variable was teachers’ overall competency in EE.

**TABLE 4:** Pearson’s correlation analysis results of entrepreneurship education teachers’ overall competency.

	OC	PT	NMT	EP	EC	PS
OC	1.00					
PT	0.754***	1.00				
NMT	0.751***	0.870***	1.00			
EP	0.702***	0.810***	0.797***	1.00		
EC	0.719***	0.820***	0.825***	0.790***	1.00	
PS	0.735***	0.844***	0.850***	0.822***	0.852***	1.00

OC, overall competency. ***Significant at the levels of 1% (one-tailed test).

**TABLE 5:** Summary of regression analysis of teachers’ total competency in entrepreneurship education.

Predictive variable		Model				
		
		β	SE	*T*-value	Tolerance	VIF
(Constant)			0.005	0.000	0.185	5.411
Professional training		0.252	0.013	19.866***	0.184	5.439
New modes of teaching		0.234	0.013	18.393***	0.264	3.795
Entrepreneurial practice		0.107	0.011	10.073***	0.221	4.520
Entrepreneurial culture		0.124	0.012	10.657***	0.178	5.619
Policy support		0.131	0.013	10.101***	0.185	5.411
**Regression model**	DW	2.001				
	*F*-value	4208.685***				
	*R*^2^	0.626				

Dependent variable: total competency of teachers in entrepreneurship education (i.e., including three dimensions at the same time). ***Significant at the levels of 1%.

The calculation results showed that the multivariate correlation coefficient R of the factors linked to teachers’ overall competency in EE was 0.791, the square of the multivariate correlation coefficient was 0.626, and the *F*-value was 4208.685, reaching the significance level of 1%. Professional training (β = 0.252, *p* < 0.001), new modes of teaching (β = 0.234, *p* < 0.001), policy guarantee (β = 0.131, *p* < 0.001), entrepreneurial culture (β = 0.124, *p* < 0.001), and entrepreneurial practice (β = 0.107, *p* < 0.001) had significant influence on the improvement of the overall competency of EE teachers, and the overall fitting effect of the equation was good. It can also be seen from the standard coefficient beta that all five factors were significant and positive.

In order to draw a more scientific conclusion, it is necessary to test whether the regression model has three major problems: multicollinearity, serial correlation, and heteroscedasticity. The multicollinearity of the model was mainly tested by the tolerance and variance inflation factor (VIF). Table 5 shows that there is no multicollinearity problem in this model. The DW (J. Durbin and G. S. Watson) value is 2.001, indicating almost no sequence correlation (Muller and Stadtmuller, 1987). In addition, observing whether the scatter diagram of standardized residuals has an obvious change rule showed that there was no heteroscedasticity problem.

### Differences in the Teachers’ Competencies in EE

With gender as an independent variable and teachers’ overall competency in EE as a dependent variable, SPSS 25.0 was used for one-way analysis of variance (ANOVA) and the least significant difference method. According to the ANOVA table output by SPSS, *F* = 4.347, *P* = 0.037 < 0.05 indicated that the difference was significant. Therefore, the empirical results showed that female EE teachers were significantly stronger than male teachers in terms of overall competency (*F* = 4.347, *p* = 0.037). In this research, female teachers accounted for 56.4% of the total teacher sample. Nowadays, both eastern and western countries are confronted with the imbalance of teacher gender structure and the gradual feminization of the teaching staff (Fu, 2000; Carrington and McPhee, 2008). With the development of the economy and society, the reform of education is bound to be promoted. In order to better balance the development of EE teachers, it is necessary to strive to realize equal rights for male and female teachers. In modern society, female entrepreneurs are considered less important than male entrepreneurs, so scholars in entrepreneurship often advocate to strengthen the support given to female entrepreneurship (Gupta et al., 2019; Hechavarria et al., 2019). However, under the perspective of teachers’ competencies, this research result showed that male teachers should also be supported.

This study showed that the overall competency of teachers aged 31–35, at the significance level of 5%, was significantly higher than of those in other age groups. That is, the growth curve for overall teacher competency reaches its peak at the second stage “31–35 years old” and then declines and becomes stable at the “36–40 years old” stage, while there is no significant difference between the “36–40 years old” and the “41 years old and above” stages. According to the research data, the largest proportion of teachers (39.1%) was under the age of 30. In our opinion, one possible reason may be that young teachers have better innovation ability and entrepreneurial potential than older teachers (Henderson and Robertson, 1999; Shao and Wang, 2019), and teachers aged 31–35 have more teaching experience and practical experience than those under 30 years old. Therefore, the overall competence of teachers aged 31–35 is significantly higher. The teachers of entrepreneurship are younger, so the stereotype against younger teachers should be abandoned, and development opportunities should be provided to teacher groups at different ages. The competency of teachers of entrepreneurship is the core factor to improving the implementation of EE and developing students’ competencies.

The empirical results showed that, at the significance level of 5%, teachers of entrepreneurship in “double first-class” universities are significantly higher than those in independent colleges in the overall competency dimension, with no significant difference from other types of universities. The overall competency of teachers of entrepreneurship in ordinary undergraduate colleges is significantly lower than that in higher vocational colleges, and the difference from other types of colleges is not obvious. In terms of overall competency, teachers of entrepreneurship in higher vocational colleges and universities score significantly higher than those of ordinary undergraduate colleges, independent colleges, and other types of colleges; however, they were not significantly different from the “double first-class” universities. To a certain extent, this shows that the current level of teachers of entrepreneurship in Chinese universities is still uneven. Upon further analyzing the data of the sample teachers, it was found that the majority of the teachers in the sample have a master’s degree (54%) and only 14.6% have a doctor’s degree. In terms of years of working experience in EE, 39.5% of teachers indicated “2 years or less” and 26.2% indicated “3–5 years”; a score of 65.7% indicated that most teachers of entrepreneurship were still beginners. In terms of teacher types, 35.3% of teachers worked as counselors, followed by non-entrepreneurial professional teachers (24%), and entrepreneurial professional teachers (16%). Therefore, in general, there are too few vocational teachers for EE in China, the training of and emphasis on part-time teachers are not enough, and their level of professionalism is low.

## Discussion

The results showed that doing well in professional training, new modes of teaching, policy guarantees, entrepreneurial culture, and entrepreneurial practice have a significant impact on the improvement of the overall competency of teachers. Among them, the regression coefficient of professional training was 0.252, ranking the first. This showed that the most important way to improve the competency of teachers was to increase the breadth and depth of the professional training of teachers. That is, the indicators “M2 – Encourages teachers to participate in entrepreneurship teacher training,” “M5 – Attaches importance to entrepreneurship education in pre-service teacher education,” and “M17 – Makes scientific career planning for the professional development of entrepreneurship teachers” have also passed empirical verification. On the one hand, professional training should pay attention to the training needs of teachers. Is it due to teachers’ reluctance or lack of knowledge and skills? On the other hand, schools’ teacher development center and other management departments should provide more professional support. Therefore, how to design a training program precisely matching the professional development needs of teachers of EE and carrying out professional training based on new Internet technologies are worth being studied in-depth.

## Conclusion

Based on our distribution of 12596 teacher questionnaires in China and the ANOVA, the influence factors of entrepreneurship teachers’ competency were found to be professional training, new modes of teaching, entrepreneurial practice, entrepreneurial culture, and policy guarantees. Our findings provide novel insights by exploring factors linked to teachers’ competencies and extending our understanding of improving the quality of EE and enrich the EE literature by adding new empirical evidence from China.

The limitation of this study is that more factors should be considered to further explore teachers’ competencies in EE and the relationship between teachers’ competencies and the quality of EE. Categories of respondents should be added to the questionnaire to explain the occupational distribution of the sample. Furthermore, further study on how to improve teachers’ competency could adopt a pretest and posttest design, preferably with a control group based on the scale developed in this paper.

## Implications

### Adopt New Modes of Teaching in Entrepreneurship Education

First, the study showed that the regression coefficient of new modes of teaching was 0.234, ranking second among factors affecting the overall competency improvement of teachers in EE. The constituent indicators are M4, M7, and M18: Teachers are encouraged to deeply integrate professional courses with EE. For sustainable entrepreneurship, Chinese universities should, according to their own characteristics and specific situation, choose an effective model. Furthermore, universities should further combine professional education and EE.

Second, the world is entering an era of innovation 3.0, characterized by the ecosystem and network ecology. Therefore, attention should be paid to the teaching method of active learning and experiential learning to cultivate talents for the innovation 3.0 era.

### Pay Attention to Teachers’ Careers in Entrepreneurship Education

Encouraging teachers to strengthen theoretical research on EE is of great significance to the teachers’ careers and the improvement of the quality of EE in colleges and universities. From 1999 to early 2019, Chinese National Knowledge Infrastructure revealed that more than 31,500 journal articles on “entrepreneurship education” were published in China. In addition, the study showed that a minimal number of teachers (14.6%) from the sample had a doctoral degree. Therefore, schools should encourage teachers of EE to pursue doctoral degrees.

It is important to give teachers enough time and space to develop their teaching competency; promote the effective connection between teachers’ career development period, growth period, and maturity period; stimulate the vitality of teachers at different ages; and meet their development needs in various aspects, so as to improve their professional competencies. Furthermore, according to the characteristics of talents of different ages, the career path of high-level teachers should be formulated and the corresponding platform for capacity improvement should be provided.

### Improve the Evaluation and Recruitment Mechanism for Teachers of Entrepreneurship Education

As observed earlier, only 39.5% of the sampled teachers had been engaged in EE for 2 years or less, that is, most of them are in the early stages of their career. However, job satisfaction (Saari and Judge, 2010) in the early career of knowledge-skilled employees had a significant negative prediction effect on turnover intention. The lowest satisfaction of sampled teachers in evaluating the current situation of EE in their schools was with respect to the number of teachers, the combination of professional and part-time teachers, scientific performance appraisal and professional title appraisal, and employment mechanism.

Therefore, it is necessary to scientifically plan the career of teachers engaged in EE and improve their evaluation mechanism, so that teachers have the time and willingness to devote themselves to EE. The empirical results showed that the regression coefficient of policy guarantees ranked third among the factors influencing the improvement of teachers’ competency in EE. Five indicators M8, M9, M15, M16, and M19 were closely related. Rasmussen’s research indicated that effective policies and actions should also be multilevel and continuous, and these policies should be embedded in all levels of universities and teachers, such as university administrators, research teams, and industrial partners (Rasmussen and Borch, 2010; Rasmussen et al., 2014). Thus, teachers’ opinions should be fully comprehended to meet their basic needs.

## Data Availability Statement

The original contributions presented in the study are included in the article/supplementary material, further inquiries can be directed to the corresponding authors.

## Ethics Statement

An ethics approval was not required as per applicable institutional and national guidelines and regulations. The informed consent of the participants was implied through survey completion.

## Author Contributions

YH and LA described and developed the review and the hypothesis, performed the analysis, interpreted the results, and formulated the main conclusions. YH and PW were involved in the data collection process. LL and ZZ formulated the study limitations and future directions for research.

## Conflict of Interest

The authors declare that the research was conducted in the absence of any commercial or financial relationships that could be construed as a potential conflict of interest.
